# Assuring the safety of AI-based clinical decision support systems: a case study of the AI Clinician for sepsis treatment

**DOI:** 10.1136/bmjhci-2022-100549

**Published:** 2022-07-14

**Authors:** Paul Festor, Yan Jia, Anthony C Gordon, A Aldo Faisal, Ibrahim Habli, Matthieu Komorowski

**Affiliations:** 1 UKRI Centre for Doctoral Training in AI for Healthcare, Imperial College London, London, UK; 2 Brain & Behvaiour Lab: Departments of Bioengineering and Computing, Imperial College London, London, UK; 3 Assuring Autonomy International Programme, University of York, York, UK; 4 Department of Computing, University of York, York, UK; 5 Department of Surgery and Cancer, Imperial College London, London, UK; 6 Institute of artificial and human intellgience, Universität Bayreuth, Bayreuth, Bayern, Germany; 7 Department of Computer Science, University of York, York, UK

**Keywords:** artificial intelligence, safety Management, decision support systems, clinical, machine Learning

## Abstract

**Objectives:**

Establishing confidence in the safety of Artificial Intelligence (AI)-based clinical decision support systems is important prior to clinical deployment and regulatory approval for systems with increasing autonomy. Here, we undertook safety assurance of the AI Clinician, a previously published reinforcement learning-based treatment recommendation system for sepsis.

**Methods:**

As part of the safety assurance, we defined four clinical hazards in sepsis resuscitation based on clinical expert opinion and the existing literature. We then identified a set of unsafe scenarios, intended to limit the action space of the AI agent with the goal of reducing the likelihood of hazardous decisions.

**Results:**

Using a subset of the Medical Information Mart for Intensive Care (MIMIC-III) database, we demonstrated that our previously published ‘AI clinician’ recommended fewer hazardous decisions than human clinicians in three out of our four predefined clinical scenarios, while the difference was not statistically significant in the fourth scenario. Then, we modified the reward function to satisfy our safety constraints and trained a new AI Clinician agent. The retrained model shows enhanced safety, without negatively impacting model performance.

**Discussion:**

While some contextual patient information absent from the data may have pushed human clinicians to take hazardous actions, the data were curated to limit the impact of this confounder.

**Conclusion:**

These advances provide a use case for the systematic safety assurance of AI-based clinical systems towards the generation of explicit safety evidence, which could be replicated for other AI applications or other clinical contexts, and inform medical device regulatory bodies.

**What is already known on this topic**

- Reinforcement learning can be applied to model and optimise the haemodynamic management of severe infections in the intensive care unit.
- Safety assessment frameworks for autonomous and semiautonomous systems are available in the safety engineering community and can be extended to the healthcare domain.

**What this study adds**

- Expert-defined scenarios can be used to assess the safety of AI-based clinical decision support systems prior to clinical deployment and compare them with human clinicians’ performance.
- Reward reshaping provides a pragmatic solution to improve reinforcement learning performance within predefined safety constraints.

**How this study might affect research, practice or policy**

- This study provides a use case for the systematic safety assurance of AI-based clinical decision support systems.
- This work could serve as a blueprint for other AI applications and inform medical device regulatory bodies.

## Introduction

Several recent publications have shed light on the pressing issue of the safety of AI-based clinical decision systems and digital technologies, for example, with a trial of an acute kidney injury alerting system showing possible harm in some contexts.^1^ Safety assurance should not be seen as a post hoc bolt-on activity. Instead, best practices from safety-critical systems engineering should be woven into the design of AI systems and should proactively lead to the generation of safety evidence for the use of the tool in its intended clinical pathway.^2^

These safety engineering concepts have been incorporated into safety assessment methodologies, such as Assurance of Machine Learning in Autonomous Systems (AMLAS).^3^ AMLAS takes a whole system approach to safety assurance. It aims to establish traceable links between the system-level hazards, risks and the safety requirements that have to be satisfied by the machine learning components. It also complements current initiatives and studies that focus on the human and organisational aspects of clinical risk management.^4 5^ See online supplemental appendix A for more detail on AMLAS. AMLAS is used here for its modular and iterative approach to safety assessment of a product over its whole lifecycle. The flexibility granted by these properties is essential in a complex context such as healthcare since safety considerations are only meaningful once scoped within the wider clinical setting.

10.1136/bmjhci-2022-100549.supp1 Supplementary data

**Figure 1:**
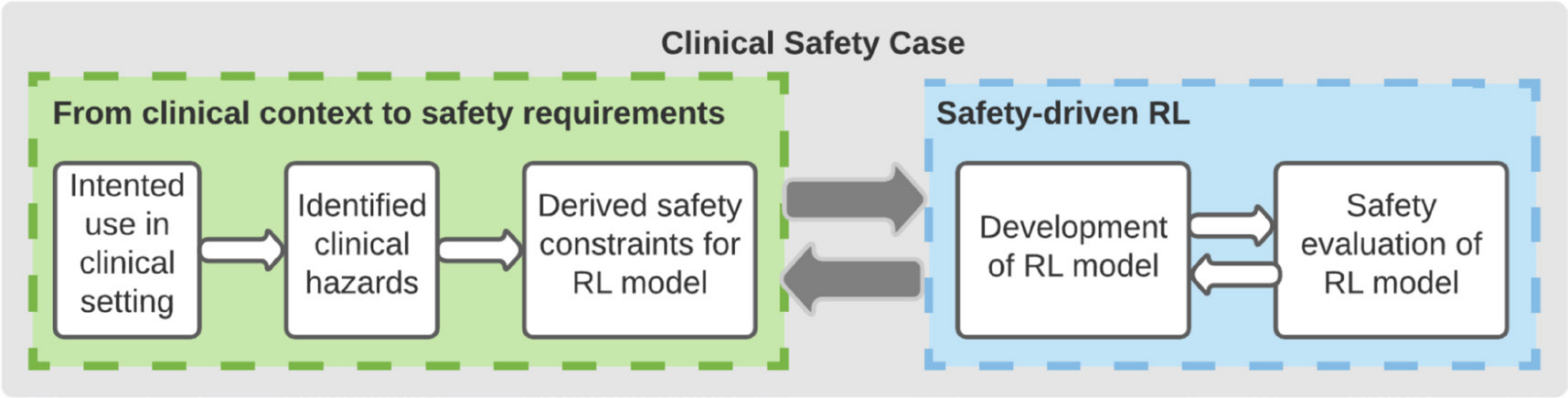
Safety assurance methodology for the AI Clinician (adapted from AMLAS^3^). The process alternates between defining safety constraints to mitigate clinical hazards in the context of use of the system (left panel) and model adjustments and evaluation against the defined constraints. AMLAS, Assurance of Machine Learning in Autonomous Systems.

In this work, we applied the principles of the AMLAS methodology to a previously published AI model built for informing the treatment of sepsis (severe infections with organ failure) as presented in figure 1.^6^

Sepsis is a common cause of hospital and intensive care unit (ICU) admission, morbidity and mortality and a major source of healthcare expenditure.^7^ A cornerstone of sepsis management includes the administration of intravenous fluids and/or vasopressors to restore a normal circulating blood volume and prevent further organ dysfunction. However, determining the correct dose and timing of these interventions is highly challenging for human doctors.^8 9^

In previous research, we developed the AI Clinician, a clinical decision support algorithm based on Reinforcement Learning (RL), capable of suggesting a dosing strategy for these two types of drugs over time and for a given individual patient.^6^ While we had generated some (retrospective) evidence of the model’s effectiveness, we had so far limited assessment of its safety.

In this work, we applied AMLAS to the AI Clinician. In particular, we identified a set of clinical hazards and potential unsafe scenarios and assessed both retrospective human clinician recorded behaviour and AI agent behaviour against these scenarios on a subset of the MIMIC-III database^10^ This created the basis for concrete safety requirements, in the form of constraints, for the AI Clinician. Then, we fed back the output of this analysis into model design and tested whether these safety requirements could be satisfied by the RL agent, as well as the impact that these additional safety requirements would have on AI model performance.

## Methods

AMLAS requires the definition and assurance of safety requirements. For autonomous or semiautonomous systems, these requirements may include a set of safety constraints, intended to limit the action space of an agent with the goal of reducing the likelihood of hazardous decisions. These constraints may trigger an inhibiting action (to prevent the transition from a safe to an unsafe state) or a correction (to return a system into a safe state).^11^

However, defining safety constraints in a clinical context such as sepsis, where there is no expert consensus and no high-performance simulation environment (where safety limits could be explored without putting patients at risk), is highly challenging. As a consequence, we used expert opinion and the literature to define a set of four undesirable clinical scenarios. Given that the action space of the AI includes fluids and vasopressors, we selected scenarios representing possible under or overdosing of these two drugs. For more information on the scenario definition process, see online supplemental appendix A.

To study the difference in the proportion of human and AI mistakes, we model them as Bernoulli random variables with hidden parameters $p_{Human}$ and $p_{AI}$, respectively. For a given scenario, among the subset of *N* patients at risk, we observe ${\hat{x}}_{Human}$ (resp. ${\hat{x}}_{AI}$) human (resp. AI) mistakes and use a z-test to test for the null hypothesis on the underlying Bernouilli distribution parameters: $H_{0}:p_{Human}=p_{AI}$. The test statistic is given by:

$$z=\frac{{\hat{p}}_{AI}-{\hat{p}}_{Human}}{\sqrt{\hat{p}(1-\hat{p})\frac{2}{N}}}$$

where ${\hat{p}}_{AI}={\hat{x}}_{AI}/N$, ${\hat{p}}_{Human}={\hat{x}}_{Human}/N$ and $\hat{p}=({\hat{p}}_{Human}+{\hat{p}}_{Human})/2$. According to the law of large numbers, when *N* is large and $H_{0}$ is true, then z∼N(0,1). Thus, p values are computed using the standard Gaussian cumulative distribution function.

Next, we analysed which patient features were associated with human clinician unsafe decisions. We trained gradient boosting models to predict whether clinicians would take an unsafe decision given the set of patient features as input. Separate models were trained for all four scenarios. We then reported the relative SHAP importance^12^ of each feature from the fitted gradient boosting model and proposed hypotheses for the significance of the most important parameters (see online supplemental appendix A for more detail).

Finally, the results of this initial safety analysis were used to refine the AI Clinician algorithm. In RL, the optimal decisions are identified as the set of actions that maximises the sum of future expected rewards.^13^ In the initial model, the reward is based on survival at 90 days following ICU admission (positive reward if the patient survived, negative reward for death). We modified the reward function of the model by systematically penalising instances where harmful decisions were taken by clinicians in the training dataset. Specifically, we added a certain amount of penalty to any decision that satisfied our predefined unsafe scenarios, so the final reward function includes both intermediate and terminal signals (see online supplemental appendix A for more detail).

We retrained the AI Clinician with this new reward function using Q-learning, a well-established model-free off-policy RL algorithm where an optimal policy is learnt from analysing trajectories of previously recorded was generated by suboptimal agents (in this case, human clinicians).^13^ We compare the proportion of unsafe decisions in each scenario for three separate agents: human clinicians (in the training data), the original AI Clinician model and the modified ‘safe’ AI Clinician. We also estimated the value of the new modified policy, using off-policy policy evaluation,^14^ and compared it with the clinicians’ policy and the original AI Clinician policy. We used bootstrapping with 2000 resamplings to generate confidence bounds on the policy value.^15^

## Results

### Output from AMLAS: definition of the four clinical scenarios

The four clinical scenarios are outlined in table 1. As detailed in Methods and online supplemental appendix A, these represent clinical situations where one or both of the drugs of interest were likely administered either insufficiently (underdosing) or excessively (overdosing). For more detail on the scenarios, see online supplemental appendix A. The subset of MIMIC-III used in this study was extracted with the same process as in ref 6 (see online supplemental appendix A for more detail).

**Table 1:** Description and rationale for the four chosen clinical scenarios

Hazardous clinical scenario	Clinical safety impact	Prevalence in MIMIC-III dataset	Safety-driven refinement of RL model	Updated safety evidence	Caveats or uncertainties
A: giving no vasopressors and low or no fluids (≤20 mL/hour) to a patient with low BP.	Sustained untreated hypotension leading to organ failure and death.^24 25^	MAP <55: 29 089/984 269 (2.9%). Clinician’s action: 15 630/29 089 (53.7%).	Add 30 points of intermediate penalty if the condition is met.	The modified ‘safe’ policy had lower rate of unsafe behaviour than original AI policy, in three scenarios and the difference was not significant in the fourth (see figure 4)	No clear threshold for defining hypotension^26^
B: giving the maximum vasopressors dose (>0.65 µg/kg/min) to a patient with high BP.	Excessive blood pressure leading to increased risk of organ failure, bleeding and stroke.	MAP >95: 118 869/984 269 (12.1%). Clinician’s action: 2986/118 869 (2.5%).			No clear threshold for defining hypertension. Some patients may have a clinical indication for high BP targets (eg, TBI).
C: giving no fluids to a patient with low BP and low CVP.	Hypotensive and likely hypovolaemic patient left untreated.	MAP≤55 and CVP≤5: 661/984 269 (0.06%). Clinicians action: 356/661 (53.8%).			Measuring the fluid volume status is very difficult. CVP is a poor proxy but the closest approximate we have available in the data.^27^ No clear threshold of CVP for defining hypovolaemia or hypervolaemia.
D: giving the maximum dose of fluids (>240 mL/hour) to a patient with normal BP, high cumulative fluid balance and high CVP.	Giving excessive fluids to a septic patient who is unlikely to be hypovolaemic is harmful, leading to fluid accumulation, known risk factor for organ failure and poor outcomes.^28 29^	MAP≥75 and cumulative balance >10 L and CVP ≥15: 9409/984 269 (1%). Clinicians action: 3517/9409 (37.4%).			

BP, blood pressure; CVP, central venous pressure (expressed in mm Hg); MAP, mean arterial pressure (expressed in mm Hg); TBI, traumatic brain injury.

### Assessment of the AI Clinician 1.0 against the four scenarios

**Figure 2:**
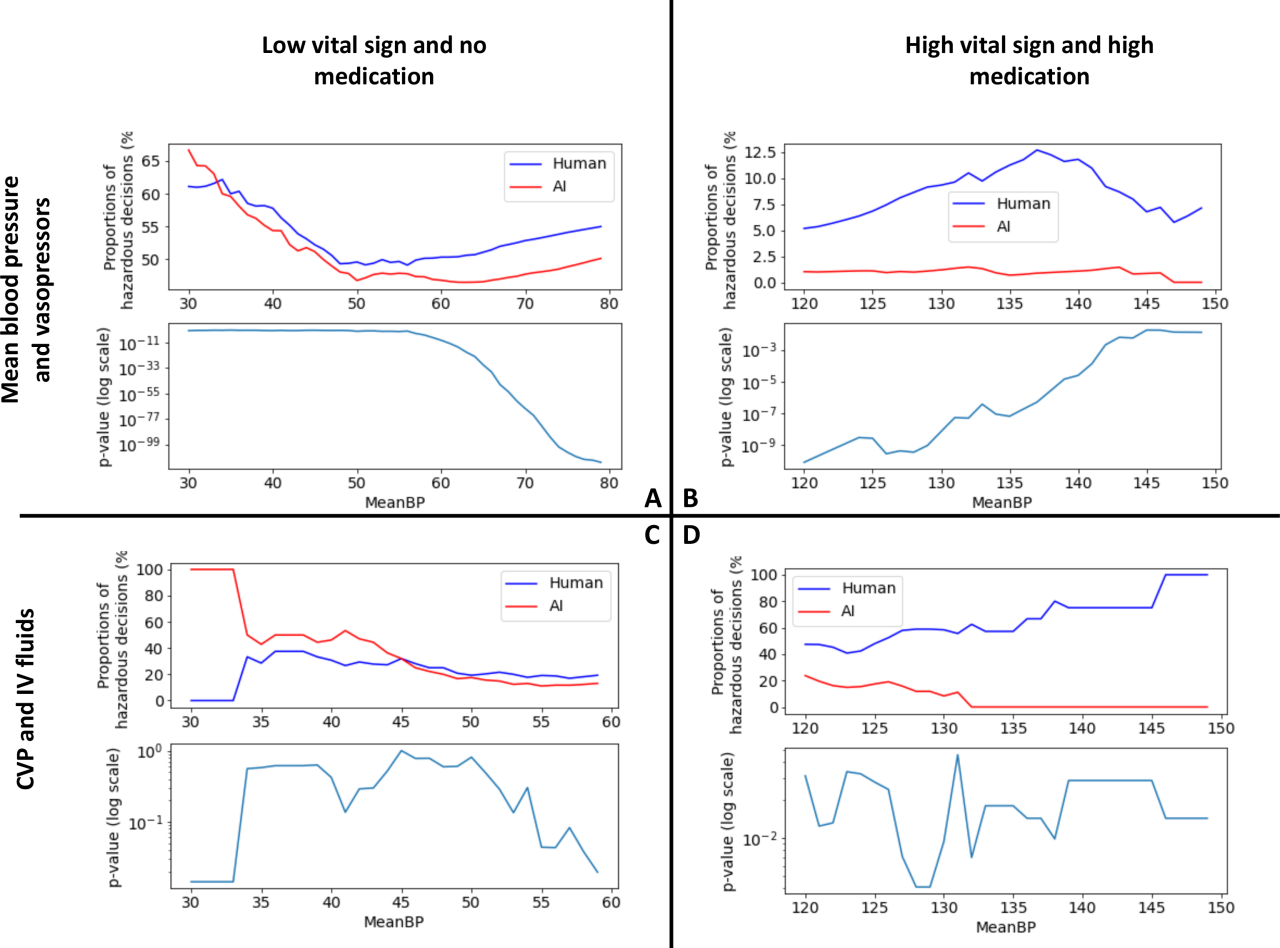
Visualisation of the differences between the proportion of human and AI unsafe decisions, and statistical significance. Each subplot corresponds to one scenario. For each scenario, tests were run across a range of blood pressure thresholds. Within each subplot, the top plot shows the variation of the number of human and AI unsafe decisions for a range of bp thresholds, and the bottom plot shows the statistical significance of this difference. The AI consistently leads to a lower number of unsafe decisions in all scenarios, except for scenario C where the difference was not statistically significant. Scenarios C and D reflect our previous study^6^ showing that the AI Clinician is more conservative in terms of fluid doses.

We studied how frequently the AI and human clinicians may contribute to one of the four hazardous clinical scenarios (figure 2). Given the lack of a clear cut-off for low and high blood pressure, the analyses were conducted on a range of thresholds. The AI consistently leads to a lower number of unsafe decisions in all scenarios (p<0.05), except for scenario C where the difference was not statistically significant.

### Analysis of patient features associated with unsafe decisions

**Figure 3:**
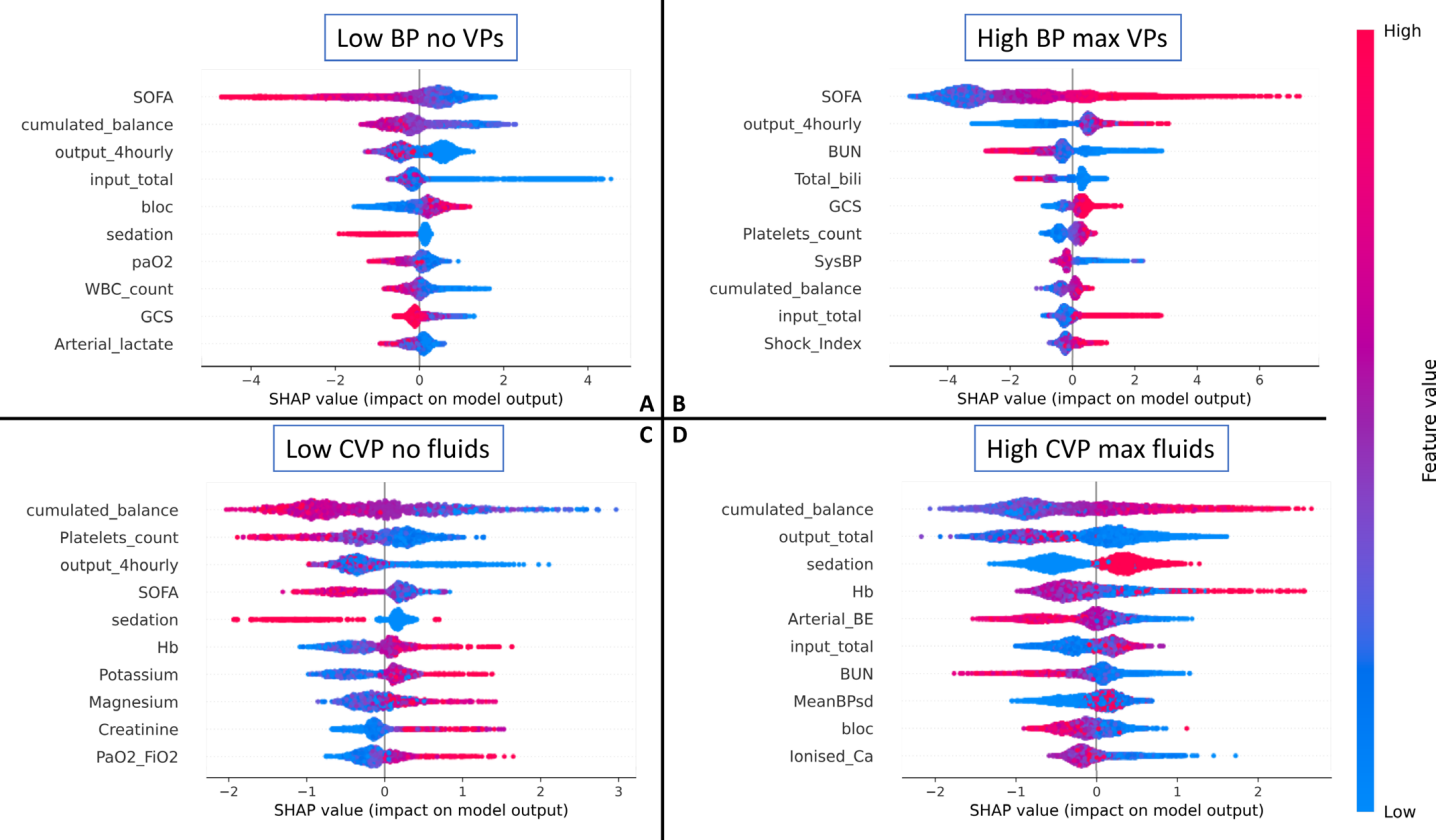
SHapley additive explanations (SHAP) relative feature importance analysis,^12^ highlighting which patient characteristics were associated with ‘unsafe’ clinician behaviour in the four scenarios. The most important features are at the top, and the less important at the bottom. On each line, a positive SHAP value indicates that the feature is associated with unsafe decisions, and the spread indicates the strength of the association. The colour indicates whether the influence stems from high or low feature values. For example, in scenario A, a low SOFA score (blue SHAP values) is associated with a higher risk (positive SHAP values) of unsafe decisions. For a glossary of terms and abbreviations, see online supplemental appendix C. SOFA, Sequential Organ Failure Assessment.

Figure 3 shows the result of the relative feature importance analysis, highlighting which patient characteristics were associated with ‘unsafe’ clinician behaviour, as defined in this study. Some hypotheses can be offered. In scenario A (low BP and no treatment initiated), a lower Sequential Organ Failure Assessment (SOFA), low cumulative fluid balance, low total fluid input, no sedation and low lactate were all associated with clinicians’ decisions labelled as unsafe. It is possible that clinicians decided to tolerate a low BP in patients who were relatively well otherwise. In scenario B (high BP and high vasopressors), a higher urine output and higher SOFA score were associated with unsafe behaviour. The high urine output could have been a consequence of an excessive blood pressure. Sicker patients (high SOFA) may have initially had a high vasopressor requirement and may have been left on high doses of vasopressors by mistake. Scenario C represents a subset of scenario A, and indeed we saw a similar pattern, where unsafe behaviour was observed in less sick patients (those with a lower SOFA). Also, the analysis highlights patients likely to be hypovolaemic (and left untreated): those with a low cumulative fluid balance and low urine output. In scenario D, a higher cumulative balance was associated with unsafe behaviour, which would be expected as the total fluid balance is correlated with previous high fluid intake. It is also interesting to note that a low urine output was associated with the unsafe behaviour of administering large volumes of intravenous fluids, which is a common decision in patients with oliguria, even though it may be harmful and fail to improve renal perfusion.

### Model retraining with additional safety constraints

**Figure 4:**
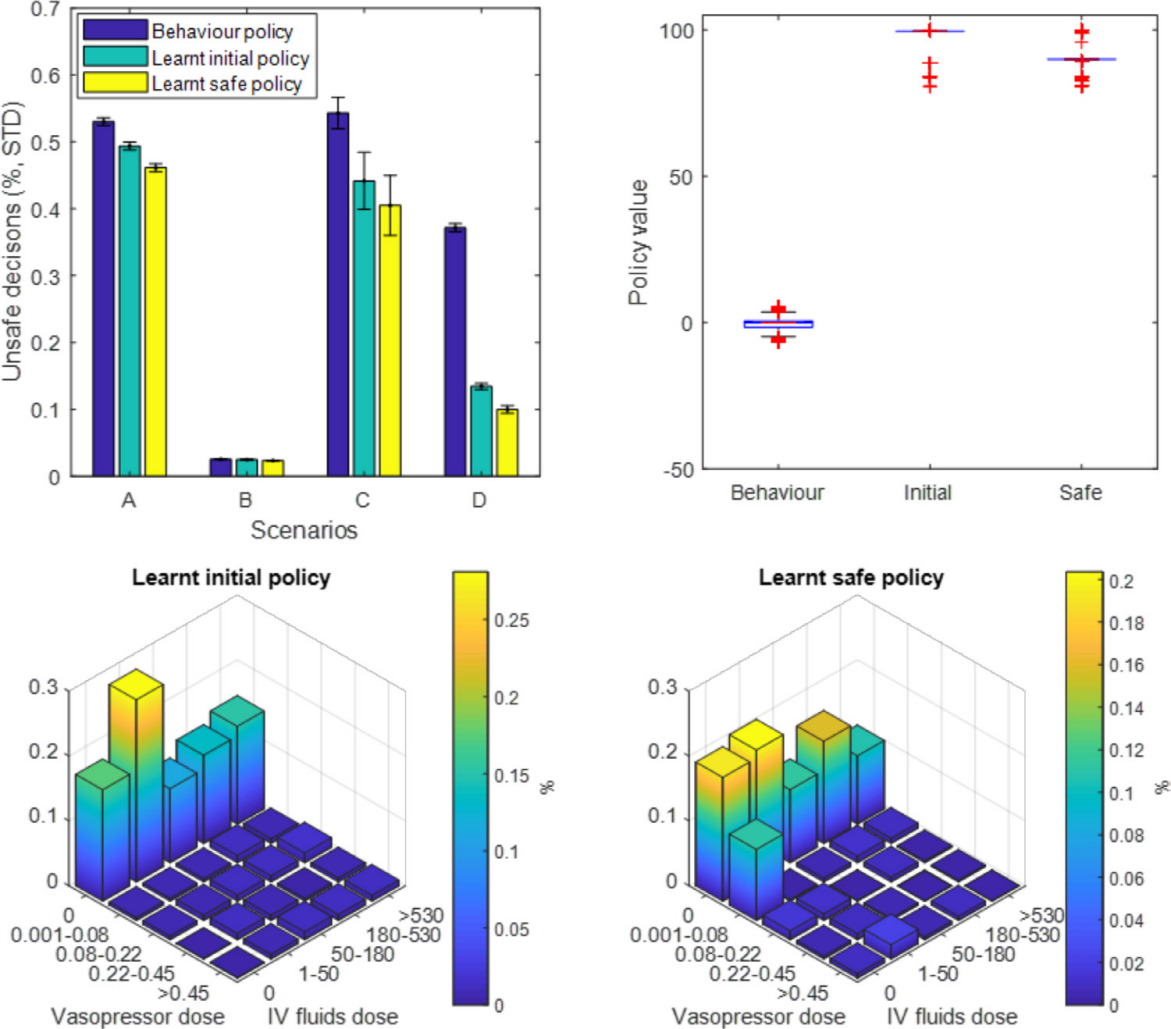
Results from the model retraining with added safety constraints. (A) Proportion of unsafe decisions in the four scenarios (see text) for three agents: human clinicians (behaviour policy), the original AI Clinician (learnt initial policy) and the modified AI Clinician (learnt safe policy). The original AI Clinician has a lower proportion of unsafe behaviour than human clinicians, while the modified ‘safe’ policy does better than the original AI Clinician. (B) Off-policy policy evaluation of the original and the modified AI Clinician policies. The value of the modified policy, with the added safety constraints, is slightly lower than the unrestricted policy (median, IQR): 90 (89.2–90) for the modified policy versus 99.5 (99.5–99.5) for the original policy, both being higher than the clinician’s (0, –1.5 to 0.6). Bottom: distribution of 25 actions for initial (C) and improved (D) policies. The safe AI policy recommends more low-dose vasopressors, likely to try and correct instances of hypotension left untreated.

The AI Clinician model was retrained with added penalties to any decision that satisfied our predefined unsafe scenarios. The four scenarios were not encountered commonly in the dataset, except scenario B (elevated blood pressure): 12.1% of the decision points corresponded to a MAP over 95 mm Hg, of which only 2.5% were labelled as ‘unsafe’ behaviour as per our definition. By trial and error, we set an additional penalty of 30 points for each of the predefined unsafe instances, which was necessary and sufficient to alter the AI policy (figure 4, see online supplemental appendix A for more detail). Figure 4A shows that the original AI Clinician had a lower proportion of unsafe behaviour than human clinicians in all four scenarios, while the modified ‘safe’ policy did better than the original AI Clinician.

Overall, human clinicians took any of the four unsafe decisions in about 2.2% of the data points in the training dataset (21 489 out of 984 269 data points). In comparison, our unaltered AI Clinician selected these decisions in 20 079 instances (a 6% relative reduction when compared with human clinicians), whereas the modified version recommended these in 18 929 instances (1.9% of the training dataset), which represents a 12% relative reduction from the clinicians’ strategy.

The off-policy policy evaluation (figure 4B) indicated that the value of the modified policy, with the added safety constraints, was only slightly lower than the original learnt policy (median, IQR): 90 (89.2–90) for the modified policy versus 99.5 (99.5–99.5) for the original policy, both much higher than the clinicians’ policy: 0 (−1.5 to 0.6). It should be kept in mind that the off-policy policy value estimation depends directly on the reward function used in the problem formulation. As such, given that the only difference of reward function between the original and adapted AI Clinician environments is the addition of penalties for unsafe behaviour, it is expected that the estimated value of the adapted AI Clinician policy is lower than that of the original policy. However, the value of the adapted policy, despite a harsher reward function, remains significantly higher than the value of the human policy in a non-penalised world.

Next, we compared the distribution of the model 25 actions for the initial (figure 4C) and modified (figure 4D) AI Clinician’s policies. The modified policy recommended more low-dose vasopressors, possibly in an effort to try and correct instances of hypotension left untreated (scenarios A and C).

## Discussion

Systematic safety assessment of AI-based clinical decision support systems is poorly codified, especially in applications where the definition of effective and safe decisions is challenging. In this study, we applied best practice in safety assurance to a complex AI system and proposed a safety-driven approach to identify regions of the action space potentially associated with preventable harm. We showed that the AI Clinician had desirable behaviour in a set of four scenarios and that we could further iteratively improve the safety of the model by adapting the reward signal without significantly compromising its performance.

To our knowledge, this work is the first successful attempt at defining and testing safety requirements for an RL-based clinical decision support system considering multiple clinical hazards and at modifying the reward function of such an agent with added safety constraints. Despite the lack of consensus on a gold standard in sepsis resuscitation, there are decisions that are ‘obviously’ dangerous, such as those we defined in this work. Given the potential harm caused by these decisions, the model will have to be *explicitly taught* to avoid them where possible. This research represents one concrete step in this direction, and we demonstrated that our modified AI was 12% less likely than human clinicians to suggest those decisions.

Regulators recognise that there is a need for better guidance on safety assurance of AI/machine learning-based systems, where this work could potentially help. The US Food and Drugs Administration has proposed the Total Product Life Cycle (TPLC) framework for assuring such systems.^16 17^ Several relevant publications provide guidance on how to systematically integrate safety concepts from the onset of system development, which could satisfy some of the key requirements of the TPLC, for example, the premarket safety assurance.^3 18^

The approach described here is necessary but not sufficient by itself. The AI Clinician V.1.0^6^ was designed as a proof-of-concept system, not meant to be used as-is in the real world. Similarly, the research presented here illustrates how RL models can be augmented with safety constraints, without substantially impairing the value of the AI policy. Thus, the commonly perceived trade-off between performance and safety is not really apparent here. If safety constraints are integrated into the AI learning process, as we show here, it is possible to enhance safety while maintaining performance. However, more in-depth technical research is needed to robustly define and assess the best way to perform reward reshaping in the context of safety assurance.

Here, we did not assess the outcomes associated with taking our custom defined safe or unsafe decisions because of methodological challenges associated with the assessment of the value and estimated outcomes of following a policy that was generated by a different agent (the problem of off-policy policy evaluation).

Another limitation is that our choice of hazardous scenarios may appear arbitrary. However, it was rationally designed following the concepts of overdosing and underdosing of the two drugs of interest, defined and refined by expert clinicians over several iterations and was constrained by the retrospective data available to us (see online supplemental appendix A for more detail). In addition, the approach is based on existing concepts of safe, warning and catastrophic states of complex systems.^11^ While this work successfully integrates four safety constraints into model learning, there remain many more loosely defined hazards, such as administrating fluid boluses to patients with (explicitly labelled) congestive heart failure, interstitial renal or pulmonary oedema, or acute respiratory distress syndrome, which should also be considered for a fully developed system. The iterative nature of the approach presented here provides a framework for the future addition of more scenarios. The penalty associated with each unsafe scenario can be tuned to reach a satisfying trade-off between model performance and the various safety constraints put in place.

We attempted to restrict our training dataset to patients with sepsis and to exclude patients with limitations and withdrawal of active treatment, as described in the original publication.^6^ As a consequence, occurrences of human underdosing or overdosing should mainly be due to external factors such as time pressure, resources or other factors that are not recorded in the dataset. However, despite our efforts to exclude these patients, some end-of-life patients in whom hypotension was left untreated will have been included. These would have: (1) artificially increased the proportion of unsafe decisions and (2) perverted correct AI model learning. Furthermore, the training data will most probably contain patients who may have had indications of unusual management. It is likely that some of the decisions labelled as unsafe were done knowingly by clinicians, for specific clinical indications. For example, patients with subarachnoid haemorrhage and cerebral vasospasm may be administered vasopressors to achieve an abnormally elevated blood pressure.

Other important components of the AMLAS methodology were not addressed in this project, including data management and model deployment testing ‘in the field’, which are also two crucial components of the TPLC. The data management process includes activities such as evaluating the data balance, accuracy and completeness, which was detailed in the original AI Clinician publication.^6^ As the aim for model deployment testing is to gather further safety evidence to support the transition towards operational evaluation and use of the system, it is best carried out following further retrospective model validation.

An emerging new avenue in the field is to augment AI models so that they can quantify their own confidence or uncertainty over their recommendations.^19^ Going forward, it may be helpful to algorithmically combine the communication of uncertainty that a system has about itself, which reflects the risk of unwanted behaviour as we have shown in other domains of risk-aware control by medical devices,^20^ with its safety features, that we have shown here.

Before widespread clinical adoption, more work is required to further assess the tool in its operational clinical context and submit it to the appraisal of bedside practitioners. Particularly, end users’ decision to act on or dismiss AI recommendations may be attached to some human-centred AI design characteristics and the degree of AI explainability.^21 22^ Human factor aspects are central in AI-based decision support systems in safety critical applications,^23^ prompting us to keep actively engineering safety into AI systems.

## Data Availability

Data are available in a public, open access repository. The MIMIC-III data set is openly available and requires applying formally for access. Access to our computer code used in this research is available by request to the corresponding author.
