# Supplementary material for: Assuring the safety of AI-based clinical decision support systems: a case study of the AI Clinician for sepsis treatment
Source: BMJ Health Care Inform. 2022 Jul 14;29(1):e100549. doi: 10.1136/bmjhci-2022-100549 (PMC9289024; doi:10.1136/bmjhci-2022-100549)
Supplement: Supplementary data [file bmjhci-2022-100549supp001.pdf]

APPENDIX A: DETAILED METHODS

AMLAS

AMLAS encapsulates a set of safety argument patterns and processes for both systematically integrating safety assurance into the machine learning model development cycle and gathering the evidence base to explicitly justify the safety of the machine learning component when integrated into systems of different levels of autonomy. The primary output of AMLAS is a safety case for the machine learning component that could be incorporated into the safety case of the wider system or service (4). AMLAS is used here for its modular and iterative approach to the safety assessment of a product over its whole lifecycle in an iterative way. The flexibility granted by these properties is essential in a complex context such as healthcare since safety considerations are only meaningful once scoped within the wider clinical setting.

DATA EXTRACTION

The subset of MIMIC-III used in this study was extracted with the same process as in (6), aiming at pulling out all ICU patients with sepsis. The final cohort slightly differs from the original paper as an update in MIMIC-III data led to an increase in number of ICU stays labelled as having sepsis from 17,083 to 20,846. The final dataset thus comprises 20,846 stays, making up for a total of 984,269 hourly recordings of patient variables and given treatment. For more details on the dataset, please refer to Supp Table 2 in appendix B.

HAZARDOUS SCENARIOS DEFINITION

In this work, we compare the performance of human and AI clinicians on the basis of how often each of them makes (or recommends) decisions that enter an unsafe scenario. Therefore, one of the main challenges of this work relies on defining what makes a decision unsafe.

In the task of cardiovascular sepsis management in the ICU where there is no gold standard for actions and where causality between actions and outcome is challenging to establish, defining unsafe scenarios for the patient is a difficult task. To tackle this question with medical expertise, the unsafe scenarios were jointly and iteratively defined by two ICU consultants (Drs Matthieu Komorowski and Anthony Gordon) and one senior ICU registrar (Dr Myura Nagendran).

Given that the purpose of this paper is to show proof of concept for safety assessment from expert knowledge, listing all possible unsafe scenarios is not necessary. The team focused on the most obvious cases of unsafe decisions related to the action space of the AI model: drug underdosing and overdosing. Other scenarios such as rapid drug dose variations were considered but set aside to focus on vasopressor/fluids underdosing/overdosing.

Festor P, *et al. BMJ Health Care Inform* 2022; 29:e100549. doi: 10.1136/bmjhci-2022-100549

In the context of sepsis, leaving a patient hypotensive most likely represents a case of underdosing of vasopressors and/or fluids, since – leaving aside heart failure - hypotension in sepsis usually results from hypovolaemia and/or vasoplegia (scenario A). Giving high vasopressors to a patient who is already hypertensive is likely harmful (scenario B). Central venous pressure can be a proxy for estimating circulating blood volume, but only with extreme values (26). We used it to define the other two scenarios, representing either hypovolaemia (scenario C) or fluid overload (scenario D). The final challenge was to set thresholds for what low/high blood pressure/fluid balance means. The final threshold values presented in table 1 are the result of the assessment of clinician experts, as well as sources from the literature, such as (27).

We further describe below the rationale behind the selected hazardous scenarios:

- **Scenario A:** Hypotension is recognised as a key contributor to sepsis mortality. Sustained untreated hypotension leads to an increased risk of organ failure and death (24,28)
- **Scenario B:** The link between hypertension and mortality in sepsis is less well characterised, but the association with cardiovascular complications, pulmonary oedema, organ damage is known.
- **Scenario C:** Central venous pressure can be a reasonable proxy for estimating circulating blood volume, but only with extreme values (26). Here, we use a low MAP in combination with a low CVP to capture patients likely to be in a hypovolaemic state, therefore requiring fluids.
- **Scenario D:** Quantifying fluid responsiveness is difficult, especially in a retrospective fashion. However, fluid accumulation of 10 litres or more is common after initial resuscitation in sepsis (29). Fluid accumulation in ICU patients is a well-known risk factor for organ failure and poor outcomes (25). The predictive positive value of high values of CVP (>12-15mmHg) for lack of fluid responsiveness is satisfactory (26). Giving high doses of fluids to such patients, when they are not hypotensive, is likely very harmful, so we included patients who were likely already fluid overloaded and still receiving large volumes of fluids.

PENALTY VALUATION AND AGENT TRAINING

Those reward signals were applied at the level of individual decision points, and all the individual decision points were then clustered into the same 752 states as in the original work (6). The rough value of the penalty was determined by clinical reasoning: the clinician experts (co-authors in the paper) agreed that any of the unsafe conditions lasting "more than a few hours" would lead to organ failure, which would likely lead to death if left untreated. (27) confirmed a mortality rate over 50% for patients with sepsis and a MAP below 55 mmHg for a duration of 2-4 hours. Equally, administering over 750 mL (3h at 245 mL/h) of IV fluids to a patient who is already fluid overloaded is likely to be very harmful, and possibly lethal. Since each patient death in our model is penalised with 100 negative points, we gave 30 points of penalty to each hour spent satisfying the dangerous criteria. This value was then fine-tuned

by trial and error to maximise the reduction in unsafe AI decisions while maintaining a high model performance.

SHAP VALUES

Developed by Lundberg to explain individual predictions (12), SHAP values were selected to report feature importance because they represent importance in a subtractive manner. Based on the difference between the model's performance with all inputs and the performance with all inputs but one, SHAP values are the same for two different features that have the same marginal contribution to the model's output.

APPENDIX B: SUPPLEMENTARY TABLES

	MIMIC-III training dataset
Unique ICUs (n)	5
Unique ICU admissions (n)	20,587
Age, years (mean (s.d.))	64.7 (16.6)
Male gender (n (%))	11,512 (66)
Premorbid status (*n* (%))<br>Hypertension<br>Diabetes<br>CHF<br>Cancer<br>COPD or RLD<br>CKD	 11,358 (55)<br>5,965 (30)<br>6,567 (31.9)<br>2,268 (11)<br>5,261 (25.5)<br>3,949 (19.2)
Primary ICD-9 diagnosis (n (%))<br>Sepsis, including pneumonia<br>Cardiovascular<br>Respiratory<br>Neurological<br>Renal<br>Others	 6,669 (32.3)<br>5,822 (28.3)<br>2,089 (10.1)<br>1,995 (9.7)<br>564 (2.7)<br>3,420 (16.6)
Initial SOFA (mean (s.d.))	7.3 (2.9)
ICU length of stay (days, median, IQR)	3 (1.6 - 6.8)
Procedures during the 72h of data collection:<br>Vasopressors (n (%))<br>Mechanical ventilation (n (%))<br>Sedation (n (%))<br>Dialysis (n (%))	 <br>6,098 (29.6)<br>10,200 (49.5)<br>8,318 (40.4)<br>1,375 (6.7)
ICU mortality (n (%))	2,120 (10.3)
90-day mortality (n (%))	4,831 (23.5)

Supp. Table 1: Description of the subset pf MIMIC-III used for this study.

	Prevalence of data	Clinicians action	Initial AI	Safe AI

	points satisfying the scenario		Clinician	Clinician
A	29,089/984,269	15,630	15,618	14,991
B	118,869/984,269	2,986	2,903	2,736
C	661/984,269	356	303	276
D	9,409/984,269	3,517	1,255	926

Supp. Table 2: Absolute values of number of unsafe decision for each policy, complement to figure 4.A.

APPENDIX C: GLOSSARY OF TERMS

- Arterial_BE: Arterial Base Excess - proxy for how acidic the blood is
- Bloc: number or hours since ICU admission
- BUN: Blood Urea Nitrogen test - measures the amount of nitrogen in your blood that comes from the waste product urea.
- GCS: Glasgow Coma Scale score - objectively describe the extent of impaired consciousness in all types of acute medical and trauma patients
- Hb: Hemoglobin count - protein that carries oxygen from the lungs to the body and CO3 from the body back to the lungs
- Ionised_Ca: Ionised calcium - most active form of calcium in the blood, measured by a serum calcium blood test
- MeanBPsd: standard deviation of the Mean Blood Pressire over the last hour
- Pa02: Partial pressure of oxygen - measure if ixygen in the arteries
- Pa02_FiO2 (P/F ratio): arterial pO2 (“P”) from the ABG (Arterial Blood Gases test) divided by the FIO2 (“F”) - the fraction (percent) of inspired oxygen that the patient is receiving expressed as a decimal (60% oxygen = FIO2 of 0.60).
- SOFA: Sequantial Organ Failure Assessment score - objective score that allows for calculation of both the number and the severity of organ dysfunction in six organ systems (respiratory, coagulatory, liver, cardiovascular, renal, and neurologic)
- SysBP: Systolic Bloor Pressure - measures of the force exerted by the heart on the walls of the arteries each time it beats (in contrast with diastolic blood pressure, the pression between heart beats)
- WBC_count: White Blood Cells count - infections increase this count, a low white blood cells count while suffering aninfection can indicate difficulty to fight it
